# Organoids containing neural-like cells derived from chicken iPSCs respond to poly:IC through the RLR family

**DOI:** 10.1371/journal.pone.0285356

**Published:** 2023-05-04

**Authors:** Masafumi Katayama, Manabu Onuma, Noriko Kato, Nobuyoshi Nakajima, Tomokazu Fukuda

**Affiliations:** 1 Biodiversity Division, National Institute for Environmental Studies, Onogawa, Tsukuba, Ibaraki, Japan; 2 Graduate School of Science and Engineering, Iwate University, Ueda, Morioka-city, Japan; Macau University of Science and Technology, HONG KONG

## Abstract

There is still much room for development in pluripotent stem cell research on avian species compared to human stem cell studies. Neural cells are useful for the evaluation of risk assessment of infectious diseases since several avian species die of encephalitis derived from infectious diseases. In this study, we attempted to develop induced pluripotent stem cells (iPSCs) technology for avian species by forming organoids containing neural-like cells. In our previous study, we established two types iPSCs from chicken somatic cells, the first is iPSCs with PB-R6F reprogramming vector and the second is iPSCs with PB-TAD-7F reprogramming vector. In this study, we first compared the nature of these two cell types using RNA-seq analysis. The total gene expression of iPSCs with PB-TAD-7F was closer to that of chicken ESCs than that of iPSCs with PB-R6F; therefore, we used iPSCs with PB-TAD-7F to form organoids containing neural-like cells. We successfully established organoids containing neural-like cells from iPSCs using PB-TAD-7F. Furthermore, our organoids responded to poly:IC through the RIG-I-like receptor (RLR) family. In this study, we developed iPSCs technology for avian species via organoid formation. In the future, organoids containing neural-like cells from avian iPSCs can develop as a new evaluation tool for infectious disease risk in avian species, including endangered avian species.

## Introduction

Induced pluripotent stem cells (iPSC) were established from somatic cells [1]. This technology allows us to avoid the use of embryos; therefore permitting the generation of pluripotent stem cells of various animals, including endangered species. Pluripotent stem cells, including iPSCs, are valuable tools since they can differentiate into various cell types, such as neural cells and hepatocytes. These differentiated cells can use in various studies, such as risk assessment, drug effects evaluation, and disease mechanisms elucidation. In human studies, pluripotent stem cells already use for various studies, such as the evaluation of drugs, pollutant risk, and risk assessment of infectious diseases [2–5]. In contrast, the application of iPSC technology to avian species is scarce. Therefore, the application of iPSC technology to avian species is still in the process of development.

In previous studies, chicken and quail somatic cells could reprogram with four to six genes [6–10]. In our previous study, chicken iPSCs efficiently established with six factors within the polycistronic reprogramming vector (PB-R6F). The PB-R6F vector contain *M3O* (MyoD-derived transactivation domain fused with *Oct3/4*), *Sox2*, *Klf4*, *c-Myc*, *Lin28*, and *Nanog* [11]. Recently, we established novel iPSCs from three endangered avian species (Okinawa rail (*Hypotaenidia okinawae*), Japanese ptarmigan (*Lagopus muta japonica*), and Blakiston’s fish owl (*Bubo blakistoni)*) using seven reprogramming factors (*M3O*, *Sox2*, *Klf4*, *c-Myc*, *Nanog*, *Lin28*, and *Klf2*) [12]. Therefore, iPSC technology has been developing for avian species as step-by-step.

Infectious diseases, such as the avian influenza virus, lead to significant economic losses in poultry farming [13]. In addition to the loss of poultry, infectious diseases have led to a dramatic reduction in the number of wild avian species, including endangered avian species. Indeed, more than 400,000 non-poultry birds, including wild species, died of avian influenza worldwide between 2021 and 2022 [14]. Therefore, assessment of the risk of infection is valuable for avian species. Encephalitis is one of the major causes of death due to infectious diseases in birds [15]. Therefore there is possible to evaluate the risk of death due to infectious diseases, such as avian influenza, if neural-like cells formed from avian iPSC. We focused on organoid formation since organoids can produce high-quality cells compared with the previous two-dimension differentiation methods. In human studies, cerebral organoids have formed from human pluripotent stem cells [16–18]. The cerebrum consists of many neural cells. Therefore, according to the organoid formation methods, we attempted to form organoids containing neural-like cells.

In this study, we attempted to develop the iPSC technology for avian species by forming organoids containing neural-like cells in chicken. Chickens have the most developed genomic information of any avian species. We therefore chose the chicken as avian species model. We first compared the nature of two types of iPSCs to select better-quality ones. Next, we generated neural-like cell-containing organoids from the iPSCs with PB-TAD-7F. Further, we confirmed that our organoids responded to poly:IC through the RIG-I-like receptor (RLR) family.

## Material and methods

### RNA-seq and differentially expressed genes (DEGs) analysis

In previous reports, we established the chicken iPSCs with PB-R6F or PB-TAD-7F reprogramming vector [11, 12]. We uploaded the iPSC-derived RNA-seq data to a public database (DDBJ; DNA Data Bank of Japan). To analyze the RNA-seq data, we used the CLC Genomic Workbench (CLC Bio, Aarhus, Denmark). To elevate the quality of those sequences, we performed the trimming. In the trimming step, we first removed the low-quality reads using CLC Genomic Workbench program. we next removed the 5-’ end, 3-end, and short sequences (shorter than 15 sequences) using CLC Genomic Workbench program. Trimmed sequence data mapped to the chicken reference genome (PRJNA698609) to retrieve gene expression data. The principal component analysis (PCA) was performed, and a heat map was created with the CLC Genomic Workbench using Transcript-Level Expression data. The plot shows the projection of the samples onto the two-dimensional space spanned by the first and second principal components of the covariance matrix. The expression levels used as input are normalized log CPM value. In this step, normalization was automatically performed using the TMM method. RNA-seq data from SRA (SRP115012 (GEO: GSE102353)) were used to compare chicken cells.

In this study, we compared the gene expression between iPSCs generated with PB-R6F and iPSCs with PB-TAD-7F, iPSCs with PB-TAD-7F and fibroblasts, and iPSCs with PB-TAD-7F and Stage X embryos. To extract differentially expressed genes (DEGs), we used the CLC Genomic Workbench program. In this study, we extracted approximately 1000 genes from DEGs in ascending order of their FDR-P values. The extracted genes were processed using the DAVID pathway analysis tool (https://david.ncifcrf.gov).

### iPSCs culture

We used chicken iPSCs with PB-TAD-7F to form organoids containing neural-like cells. The Basic medium for chicken iPSCs with PB-TAD-7F was the KAv-1. The composition of KAv-1 for avian iPSCs was as follows: alpha-MEM containing 5% FBS, 5% chicken serum, and 1% antibiotic–antimycotic mixed solution; also, 1% nonessential amino acids (Wako Pure Chemical Industries), and 2 mM glutamic acid were added (Nacalai Tesque, Kyoto, Japan). As a supplement to the medium, we further used 1,000× human LIF (125–05603; Wako Pure Chemical Industries), 4.0 ng/ml basic FGF (064–04541; Wako Pure Chemical Industries), 0.75 μM CHIR99021 (034–23103; Wako Pure Chemical Industries), and 0.25 μM PD0325901 (163–24001; Wako Pure Chemical Industries). This study cultured chicken iPSCs with PB-TAD-7F on MEF feeder cells at 37°C under 5% CO_2_.

### Formation of organoids containing neural-like cells

To form organoids containing neural-like cells, we used the chicken iPSCs with PB-TAD-7F. Chicken iPSCs with PB-TAD-7F were seeded in a V-bottom ultra-low attachment plate (PrimeSurface, MS-9096V) to form organoids. Chicken iPSCs were seeded at a density of 1×10^4^ cells per well. We enzymatically treated the ES cells (StemPro Accutase Cell Dissociation Reagent (A1110501, Thermo Fisher Scientific) and suspended them as single cells. Each dish’s total number of cells was counted using an automated cell counter (Thermo Fisher). Cell viability was assessed by staining the cells with a trypan blue solution. In this stage, the composition of the culture medium was as follows: alpha-MEM containing 2.5% FBS, 2.5% chicken serum, 5% KSR (KnockOut^™^ Serum Replacement, 10828028, Thermo Fisher), 1% antibiotic–antimycotic mixed solution, 1.15% nonessential amino acids (Wako Pure Chemical Industries), 2.3 mmol/L L-Alanyl-L-Glutamine Solution (016–21841; FUJIFILM Wako), and 1.15 mmol/L Sodium Pyruvate Solution (190–14881; FUJIFILM Wako). As supplements to the medium, we used 5 μM SB431542 (192–16541; FUJIFILM Wako), 2.5 μM IWR-1-endo (I0161-5MG; Sigma-Aldrich), and 10 μM Y27632 (036–24023; FUJIFILM Wako).

Next, we used a spheroid formation culture container dish to mature organoids containing neural-like cells. At this stage, the composition of the culture medium was as follows: DMEM/F12 (048–29785; FUJIFILM Wako), 1% N2 Supplement with Transferrin (Holo) (141–08941; FUJIFILM Wako), and 1% antibiotic–antimycotic mixed solution. In this study, we examined the concentration of FBS and chicken serum. Fig 3b shows those concentrations.

### RNA extraction and quantitative PCR (qPCR)

Total RNA was extracted from the cultured cells using NucleoSpin^®^ RNA (740955.50; MACHEREY-NAGEL, Düren, Germany). After measuring the concentration of total RNA from cultured cells, we synthesized complementary DNA (cDNA) using the PrimeScript^™^ RT Reagent Kit with gDNA Eraser (Perfect Real Time) (RR047A; Takara Bio, Shiga, Japan). The quantitative real-time PCR was performed in a 12.5-μL final volume containing 2× KOD SYBR^®^ qPCR Mix (QKD-201; TOYOBO, Osaka, Japan), 12.5 ng of cDNA, 0.5 μL of Rox, 0.3 μM each primer, and distilled water (up to 12.5 μL). S1 Table in S1 File shows primer sequences. Quantitative real-time PCR was performed using the Applied Biosystems 7300 system (Thermo Fisher Scientific). The cycling conditions were as 98°C for 2 min (initial denature), 98 °C for 10 seconds (denaturing), 58°C for 10 seconds (annealing), and 68°C 32 seconds (extension) for 45 cycles. To normalize the target gene expressions, we used *GAPDH*.

### Immunofluorescent staining

The organoids were embedded in optimal cutting temperature compound (Sakura Finetek Japan, Tokyo, Japan), frozen in liquid nitrogen, and stored at −80 °C until use. Cryosections measuring 10 μm in thickness were prepared using a cryostat and air-dried for 30 min at room temperature. After washing thrice with PBS, the sections were incubated with PBS containing 1% BSA for 1 h. After blocking with 1% BSA, the sections were incubated with an anti-Tuj1 antibody (anti-beta-III tubulin (TuJ-1), R&D Systems, Minneapolis, MN, US, cat no. 55461211) overnight. The sections were then washed thrice with PBS and incubated with a secondary antibody (goat anti-mouse IgG, Alexa Fluor 568, cat no. A11004, dilution 1:200) and Cellstain- DAPI solution (DOJINDO) for 1 h.

### Exposure to poly:IC

Before poly:IC exposure, we cut the formed organoid with ophthalmologic scissors to approximately one-fourth the size under a stereoscopic microscope due to the reduction of variety of sizes. The organoids were exposed to 5 μg/mL or 50 μg/mL poly I:C (polyinosinic-polycytidylic acid sodium salt) (4287/10; R&D Systems, Minneapolis, MN) for 6 h. We randomly chose nine-pieces from each dish (exposure of 0 μg/mL, 5 μg/mL, 50 μg/mL poly:IC) from a total of 36 pieces of organoids. During preculture and poly I:C exposure, the cells were cultured in DMEM. The DMEM (043–30085; FUJIFILM Wako Pure Chemical Industries) medium contained 10% FBS (SH30396.03; Cytiva) and 1% Penicillin-Streptomycin-Amphotericin B Suspension (×100) (Antibiotic-Antimycotic Solution) (161–23181; FUJIFILM Wako Pure Chemical Industries). Cells were cultured at 37°C in 5% CO_2_.

### Statistical analysis

In this study, we first tested whether our dataset was normally distributed using the chi-square test. Some data did not show a normal distribution (S2 Table in S1 File). Therefore, we unified the non-parametric analysis used in this study. Since nonparametric analysis can perform regardless of whether normal distribution or not. To compare the three groups, we used the steal-Dwass test. This test is a non-parametric version of the Tukey-Kramer test (Figs 4, 5a, 5b, 6 and S2 Fig in S1 File). As shown in Fig 5c, we used the Mann–Whitney U test to compare the two groups. This test is also a non-parametric analysis. Statistical differences are indicated by *(*P*<0.05), **(*P*<0.01). We used the statistical analysis software Statcel3 to perform data analyses (Statcel-The Useful Addin Forms on Excel-3rd edition, OMS Publishing, Higashi-Kurume, Tokyo, Japan).

## Results

### Cellular characteristics comparison of the two types of established chicken iPSCs

**Fig 1:**
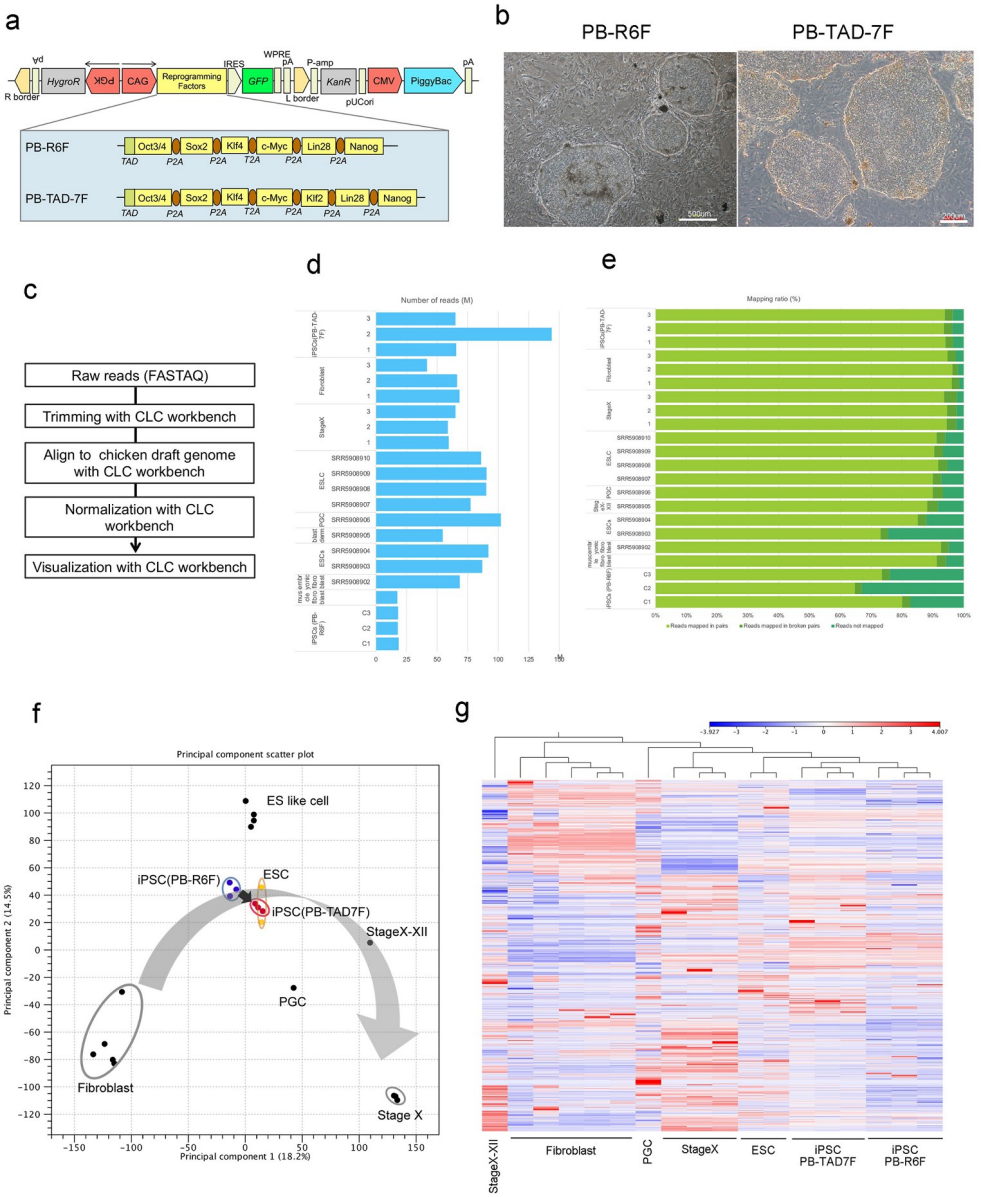
Comparison of chicken iPSCs with PB-R6F and PB-TAD-7F. a: Structure of reprogramming vector. b: Images of chicken iPSCs transfected with PB-R6F and PB-TAD-7F. Left: chicken iPSCs with PB-R6F; right: chicken iPSCs with PB-TAD-7F. The bars represent 500 μm (PB-R6F) and 200 μm (PB-TAD-7F). c: Workflow of RNA-seq analysis. d: Read numbers of chicken iPSCs with PB-R6F, iPSCs with PB-TAD-7F, fibroblasts, stage X cells, ESCs, PGCs, blastoderm cells, and ES-like cells. e: Mapping ratio of each sample. Yellow-green bars: reads mapped in pairs; green bars: reads mapped in broken pairs; blue-green bars: reads not mapped. f: PCA with profiling of chicken iPSCs with PB-R6F, iPSCs with PB-TAD-7F, chicken fibroblasts, and chicken embryo stage X with SRP115012 (GEO: GSE102353). g: Dendrogram and heat map analysis with profiling of chicken iPSCs with PB-R6F, iPSCs with PB-TAD-7F, fibroblasts, stage X cells, ESCs, PGCs, blastoderm cells, and ES-like cells.

Our previous study established two types of chicken iPSCs [11, 12]. In the first report, we established chicken iPSCs using a PB-R6F reprogramming vector [11]. The PB-R6F reprogramming vector contained a transactivation domain fused *to Oct3/4*, *Sox2*, *Klf4*, *c-Myc*, *Lin28*, *and Nanog* (Fig 1a). Next, we used the PB-TAD-7F reprogramming vector to establish chicken iPSCs [12]. The PB-TAD-7F reprogramming vector contained a transactivation domain fused *to Oct3/4*, *Sox2*, *Klf4*, *c-Myc*, *Lin28*, *Nanog*, and *Klf2* (Fig 1a). In previous studies, we did not compare the characteristics of iPSCs generated using PB-R6F and PB-TAD-7F. To form neural-like organoids, we needed to choose higher-quality cells; therefore, we compared the cellular characteristics of the two types of iPSCs (Fig 1b).

To compare the cellular characteristics of these two types of iPSCs, we performed transcriptomic analysis (Fig 1c). In this study, we compared the two types of iPSCs, chicken stage X cells, fibroblasts, ESCs, PGC, StageX-XII cells, and ES-like cells. We obtained gene expression profiles from the public databases of ESCs, PGC, StageX-XII cells, ES-like cells, and fibroblasts [9]. Fig 1d and e show the read numbers of these cells and the mapping ratio. As a result of the principal component analysis (PCA), our two established iPSCs mapped near the ESCs, compared with other cells, such as fibroblasts and Stage X cells (Fig 1f). In particular, iPSCs with PB-TAD-7F were closer to chicken ESCs than iPSCs with PB-R6F (Fig 1f). In addition to PCA, iPSCs with PB-TAD-7F were closer to chicken ESCs than iPSCs with PB-R6F in the clustering analysis (Fig 1g). Therefore, the quality of iPSCs with PB-TAD-7F would be higher than that of iPSCs with PB-R6F.

**Fig 2:**
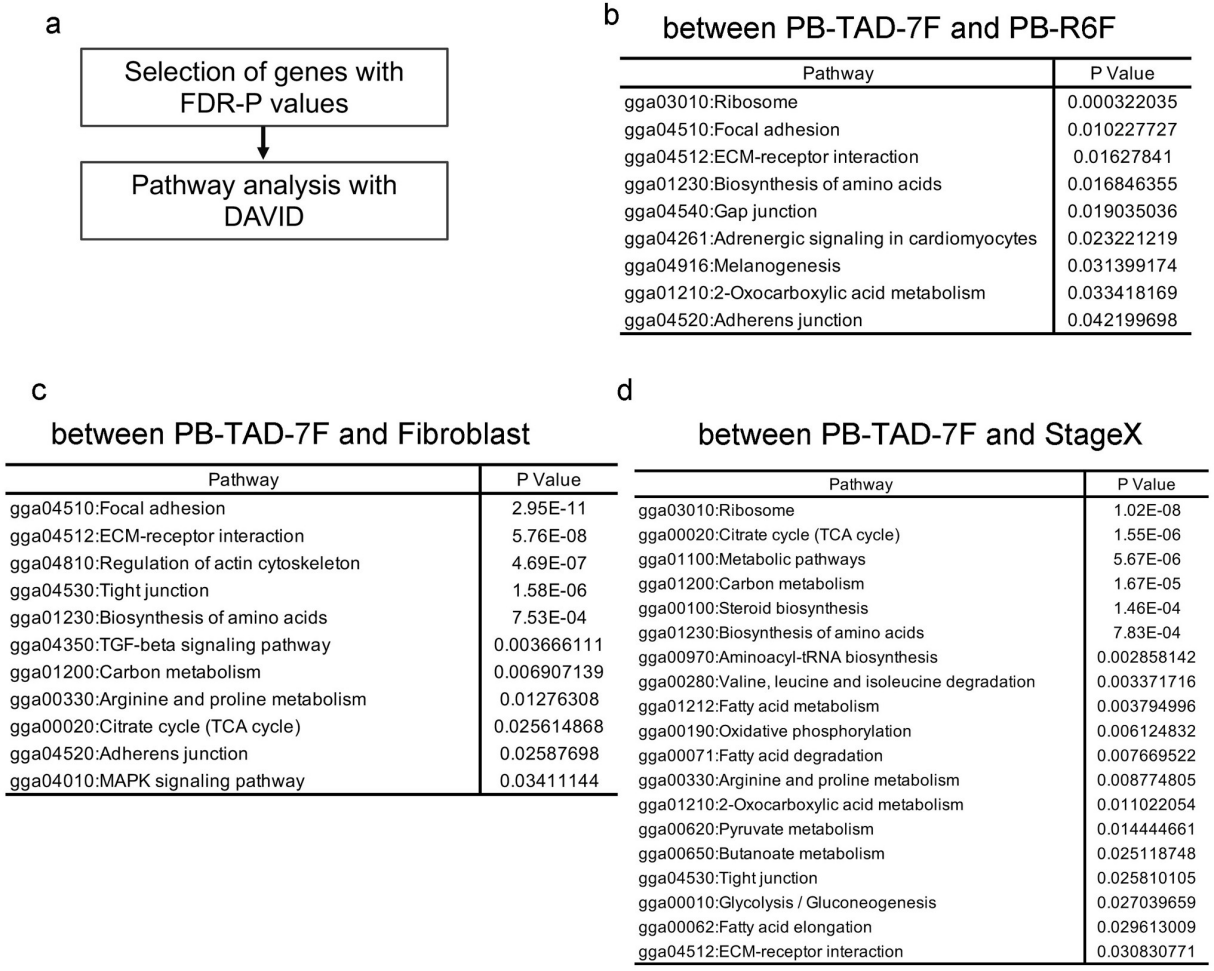
Candidate pathways with the DAVID pathway analysis tool. Workflow of candidate pathway analysis (a). List of candidate pathways between chicken iPSCs with PB-TAD-7F and chicken iPSCs with PB-R6F (b), chicken iPSCs with PB-TAD-7F and fibroblasts (c), and chicken iPSCs with PB-TAD-7F and StageX (d).

To understand more detailed differences in the characteristics of those iPSCs, we performed differentially expressed gene (DEGs) analysis. In this analysis, we attempted to identify a candidate pathway for the critical difference between iPSCs derived from PB-R6F and iPSCs derived from PB-TAD-7F. We extracted approximately 1000 genes from DEGs in ascending order of FDR-P values between iPSCs with PB-R6F and iPSCs with PB-TAD-7F. Using those genes, we searched for candidate pathways using the DAVID pathway analysis tool. Fig 2a shows the candidate pathways. We listed cellular adhesion and cellular contact-related pathways as candidate pathways between iPSCs with PB-R6F and PB-TAD-7F (Fig 2a). In this analysis, we also attempted to identify the candidate pathway for the critical difference between iPSCs with PB-TAD-7F and fibroblasts, and iPSCs with PB-TAD-7F and Stage X embryos. Similar to the candidate pathway between iPSCs with PB-R6F and PB-TAD-7F, we listed as candidate pathways the cellular adhesion and cellular contact-related pathways between iPSCs with PB-TAD-7F and fibroblasts (Fig 2b). Therefore, we considered that cellular adhesion and cellular contact-related pathways might be critical for evaluating the quality of chicken iPSCs.

Although we considered that iPSCs with PB-TAD-7F would be higher quality than iPSCs with PB-R6F, a large distance existed between iPSCs with PB-R6F and Stage X in the PCA compared with the distance between iPSCs with PB-TAD-7F and ESCs (Fig 1f). We listed metabolism-related as the critical difference pathway between iPSCs with PB-TAD-7F and Stage X (Fig 2c). These results may be helpful for improving the quality of chicken iPSCs.

### Formation of organoids containing neural-like cells with PB-TAD-7F chicken iPSCs

**Fig 3:**
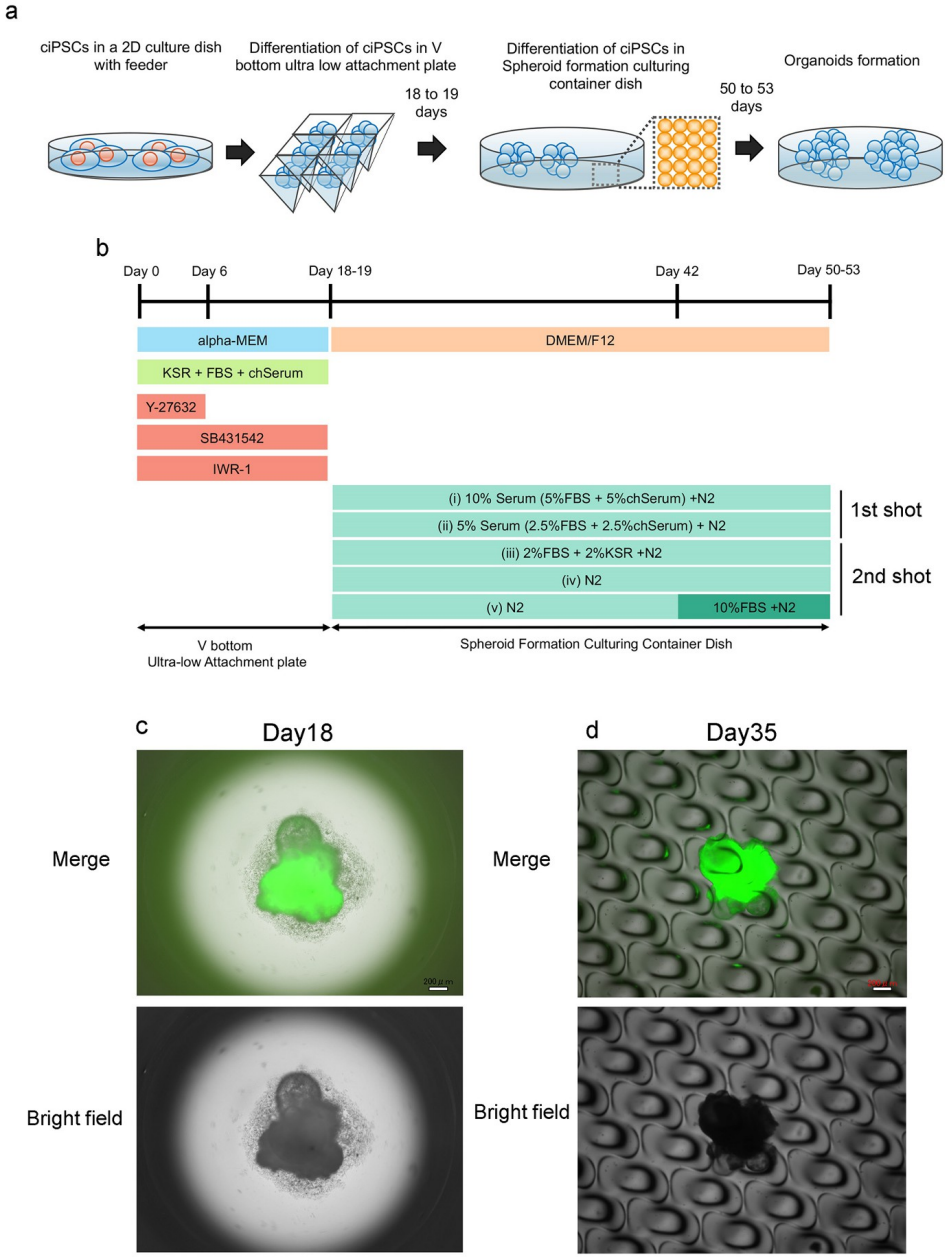
Formation of chicken iPSCs with PB-TAD-7F derived organoids containing neural-like cells. a: Schematic representation of the formation of organoids containing neuron-like cells. b: Formation flow of chicken iPSCs with PB-TAD-7F derived organoids containing neural-like cells. c, d: Morphology of organoids containing neural-like cells on days 18 (c) and 35 (d). Upper panels are merged images of bright-field and green fluorescence, and lower images are bright-field images. Scale bars: 200 μm.

In this study, we chose chicken iPSCs with PB-TAD-7F to form organoids since their quality would be higher than that of iPSCs with PB-R6F. A schematic timeline for the formation of organoids containing neural-like cells is shown in Fig 3a. As shown in Fig 3b, we attempted to form organoids containing neuron-like cells. Chicken iPSCs with PB-TAD-7F were seeded in each V-bottom Ultra-low attachment 96 well plates at a density of 1×10^4^ cells per well. Cell clusters appeared in iPSCs seeded in all wells (Fig 3c). We collected these cell clusters from an ultra-low attachment 96 well plate in a spheroid formation culture container dish. Under these conditions, we matured these cell clusters into organoids containing neural-like cells (Fig 3d).

**Fig 4:**
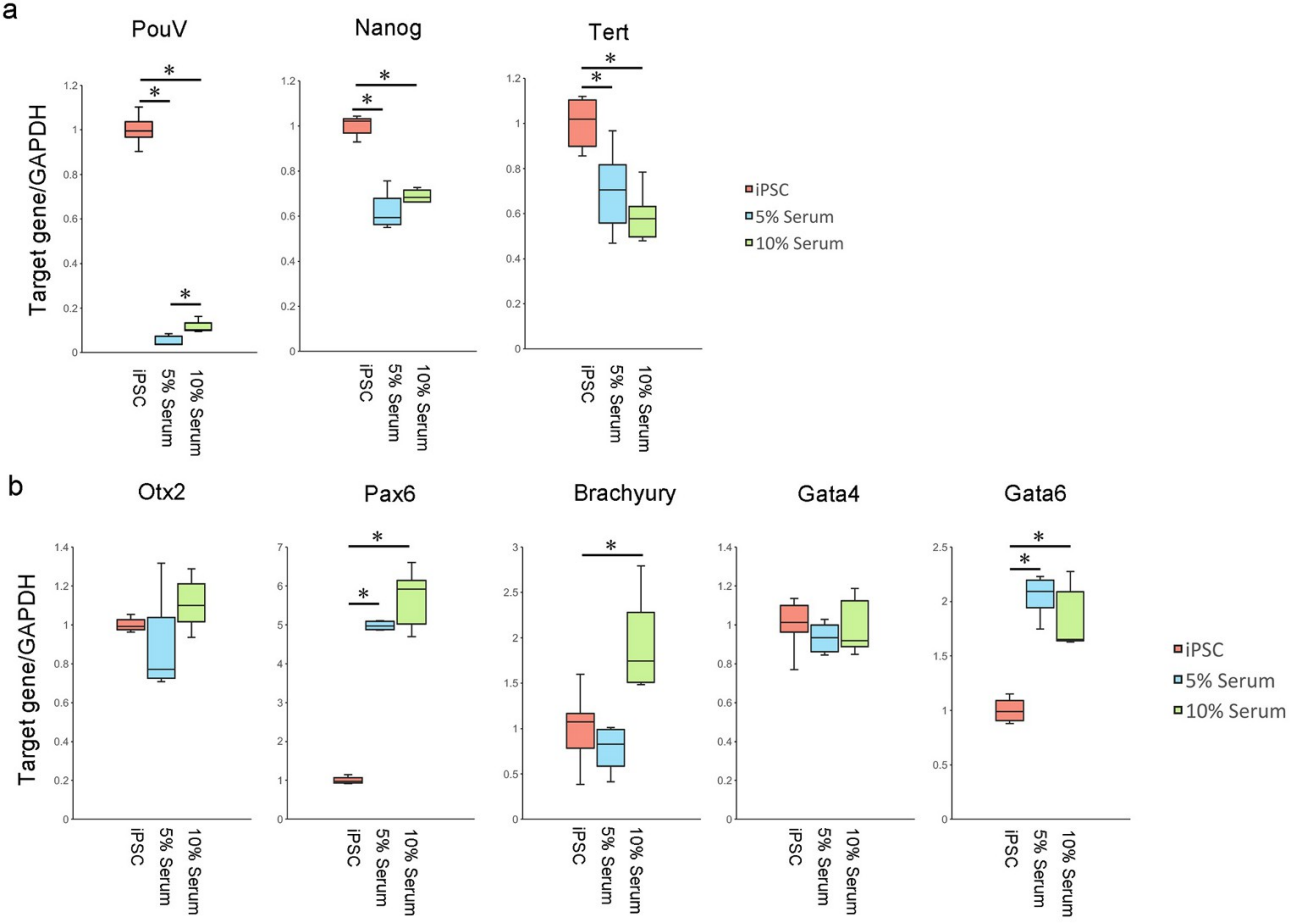
Gene expression of chicken iPSC with PB-TAD-7F organoids containing neural-like cells with 5% and 10% serum medium for maturation. a: *PouV*, *Nanog*, and *Tert* genes expressed in chicken iPSC with PB-TAD-7F, organoids containing neural-like cells in 5% serum medium for maturation, and in 10% serum medium for maturation. Centerlines of box plots indicate medians; box limits indicate 25th and 75th percentiles. n = 6. * *P*<0.05. Gene expression was quantified relative to the *GAPDH* internal control. The iPSCs level was set to 1.0. b: *Otx2*, *Pax6*, *Brachyury*, *Gata4*, and *Gata6* genes expressed in chicken iPSC with PB-TAD-7F, organoids containing neural-like cells in 5% serum medium for maturation, and in 10% serum medium for maturation. Centerlines of box plots indicate medians; box limits indicate 25th and 75th percentiles. n = 6. * *P*<0.05. Gene expression was quantified relative to the *GAPDH* internal control. The iPSCs level was set to 1.0.

Serum was a critical factor for the self-renewal of avian iPSCs in our previous study [11, 12]. In the first step of organoid maturation, we used 5% or 10% serum to mature organoids containing neural-like cells between days 18 and 53 (Fig 3b). Pluripotency-related gene expression decreased in organoids containing neural-like cells in these media (Fig 4a). Therefore, our chicken iPSCs could be differentiated from the pluripotent state in these media. Next, we analyzed the marker gene expression of the neural cells (*Otx2* and *Pax6*), mesoderm (*Brachyury*), and endoderm (*Gata4* and *Gata6*). Although the expression of *Pax6* was significantly increased from iPSCs in differentiated cells with 5% or 10% serum medium, *Otx2* expression maintained the iPSCs levels in differentiated cells with 5% or 10% serum medium (Fig 4b). *Gata4* did not increase, while *Gata6* expression and *Brachyury* expression were significantly increased in differentiated cells with 5% or 10% serum medium (Fig 4b). Based on these results, we concluded that 5% or 10% serum medium would not be sufficient to produce mature organoids containing neural-like cells.

**Fig 5:**
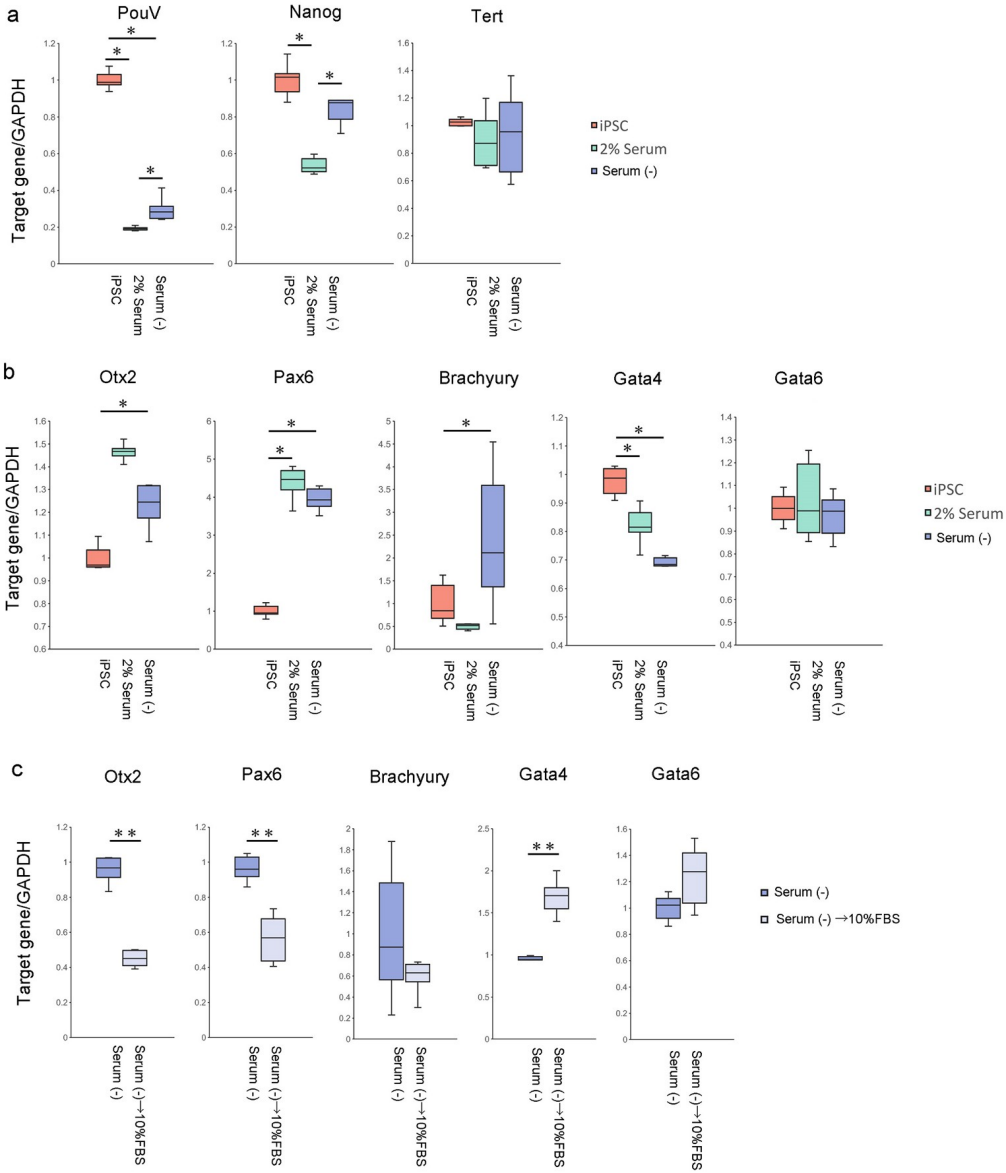
Genes expression of chicken iPSC with PB-TAD-7F, organoids containing neural-like cells with 2% serum or serum-free medium for maturation. a: *PouV*, *Nanog*, and *Tert* genes expressed in chicken iPSC with PB-TAD-7F, organoids containing neural-like cells in 2% serum medium for maturation, and organoids containing neural-like cells in serum-free medium for maturation. Centerlines of box plots indicate medians; box limits indicate 25th and 75th percentiles. n = 6. * *P*<0.05. Gene expression was quantified relative to the *GAPDH* internal control. The iPSCs level was set to 1.0. b: *Otx2*, *Pax6*, *Brachyury*, *Gata4*, and *Gata6* genes expressed in chicken iPSC with PB-TAD-7F, organoids containing neural-like cells in 2% serum or serum-free medium for maturation. Centerlines of box plots indicate medians; box limits indicate 25th and 75th percentiles. n = 6. * *P*<0.05. Gene expression was quantified relative to the *GAPDH* internal control. The iPSCs level was set to 1.0. c: *Otx2*, *Pax6*, *Brachyury*, *Gata4*, and *Gata6* genes expressed in organoids containing neural-like cells in serum-free medium for maturation, and organoids containing neural-like cells in serum-free medium to 10% serum medium for maturation. Centerlines of box plots indicate medians; box limits indicate 25th and 75th percentiles. n = 6. ** *P*<0.01. Gene expression was quantified relative to the *GAPDH* internal control. The maturation expression of neural-like organoids in a serum-free medium were 1.0.

Although we used the 5% or 10% serum to mature organoids containing neural-like cells, formed organoids were insufficient to mature in this study. To improve the quality of the organoids, we next tried to mature them in serum-free or low-serum medium since the serum-free medium is efficiently different from neural cells from pluripotent stem cells in human studies [17, 18]. Therefore, we hypothesized that serum concentrations would be critical for the maturation of organoids containing neural-like cells from chicken iPSCs. In the second step of organoid formation, we matured organoids in 2% FBS with 2% KSR or serum-free medium (Fig 3b). Although *Tert* gene expression maintained iPSCs expression in organoids containing neural-like cells, *PouV* and *Nanog* mRNA expression significantly decreased in the formed organoids compared with iPSCs (Fig 5a). Therefore, iPSCs would be differentiated from the pluripotent state in those media. *Oxt2* and *Pax6* expression was higher in the formed organoids than in chicken iPSCs. Therefore, those organoids were closer to neural cells compared with organoids of maturation with 5% or 10% serum media (Fig 5b). In contrast to *Oxt2* and *Pax6*, *Gata4* expression was significantly decreased in organoids containing neural-like cells compared to iPSCs (Fig 5b). The *Gata6* gene was similar in expression between iPSCs and organoids containing neural-like cells (Fig 5b). Based on these results, serum-free and low-serum media would improve the quality of organoids containing neural-like cells.

As additional information, we changed the medium from serum-free to 10% FBS-containing medium during the maturation of the organoids (Fig 3b iv, v); *Oxt2* and *Pax6* expression decreased in 10% FBS medium compared to that in serum-free medium (Fig 5c). These results indicate that serum-free and low-serum media would be helpful for improving the quality of organoids containing neural-like cells.

### Stimulation of organoids containing neural-like cells with poly:IC

Fibroblasts are easily obtained from skin and muscle, and their cells are easy to handle for cell culture; therefore, fibroblasts are useful bioresources for infectious disease studies, including the innate immune response of chickens [19–21]. To evaluate whether organoids containing neural-like cells can use the innate immune response of chicken in the neural cell, we expose the poly:IC. Poly:IC is an analog of viral RNA; this analog has been used in many studies to stimulate the RLR and Toll-like receptor (TLR) pathways [20, 22–25]. *MDA5*, *LGP2*, and *TLR3* are important sensors for virus recognition to stimulate innate immune responses in chickens [23, 26]. This study focused on whether or not organoids containing neural-like cells recognize poly:IC through the RLR family.

**Fig 6:**
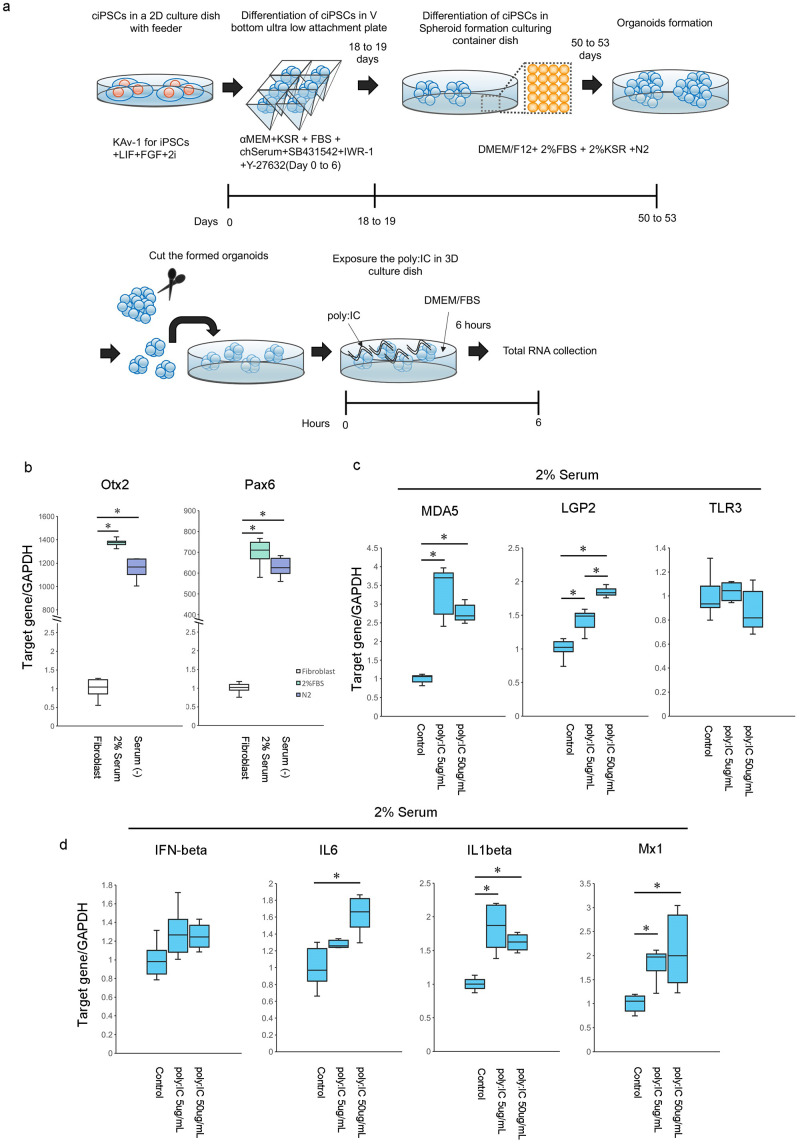
Exposure of poly:IC to organoids containing neural-like cells with 2% serum medium for maturation. a: Schematic timeline of organoid exposure to poly: IC. b: *Otx2* and *Pax6* genes expressed in chicken fibroblasts, organoids containing neural-like cells in 2% serum or serum-free medium for maturation. Centerlines of box plots indicate medians; box limits indicate 25th and 75th percentiles. n = 6. * *P*<0.05. Gene expression was quantified relative to the *GAPDH* internal control. The fibroblast expression was 1.0. c: Gene expression of *MDA5*, *LGP2*, and *TLR3* in chicken organoids containing neural-like cells in 2% serum medium for maturation after 6 h of poly:IC exposure. The left bars show control, the middle bars show exposure to poly:IC 5 μg/mL, and the right bars show exposure to poly:IC 50 μg/mL. Centerlines of box plots indicate medians; box limits indicate 25th and 75th percentiles. n = 6. * *P*<0.05. Gene expression was quantified relative to the *GAPDH* internal control. The control expression was set at 1.0. d: Gene expression of *IFN-beta*, *IL-6*, *IL-1 beta*, and *Mx1* in chicken organoids containing neural-like cells with 2% serum medium for maturation after 6 h of poly:IC exposure. The left bars show control, the middle bars show exposure to poly:IC 5 μg/mL, and the right bars show exposure to poly:IC 50 μg/mL. Centerlines of box plots indicate medians; box limits indicate 25th and 75th percentiles. n = 6. * *P*<0.05. Gene expression was quantified relative to the *GAPDH* internal control. The control expression was set at 1.0.

We generated organoids containing neural-like cells in 2% FBS with 2% KSR or serum-minus medium for maturation in this study. To expose the poly:IC, we used organoids in 2% FBS with 2% KSR medium for maturation (Fig 6a) since these organoids expressed higher levels of *Otx2* and *Pax6* than organoids in serum-free medium (Fig 6b). We confirmed that the expression of Tuj1 (a neural cell marker) in organoids matured with 2% FBS and 2% KSR medium on day 36 (S1 Fig in S1 File). In this study, although *TLR3* expression did not change after 6 h of exposure to poly:IC, *MDA5* and *LGP2* expression increased in organoids in a 2% serum medium with poly:IC exposure after 6 h (Fig 6c). Next, we evaluated the expression of downstream genes in the organoids. We observed that *IL-6*, *IL-1 beta*, and *Mx1* gene expression were significantly increased after poly: IC exposure (Fig 6d). Based on these results, we concluded that the RLR family of organoids containing neural-like cells are functional for recognizing poly:IC.

## Discussion

iPSCs are useful bioresources because of their ability to differentiate into various cell types. In human studies, various differentiated cells have been established from iPSCs, including neural cells and hepatocytes [27, 28]. In contrast to human studies, avian stem cell research still has a large room for investigation. To the best of our knowledge, this is the first report on the formation of organoids containing neural-like cells from chicken iPSCs. In this study, we developed avian iPSCs technology by forming organoids containing neural-like cells from chicken iPSCs.

We compared the nature of chicken iPSCs with PB-R6F and PB-TAD-7F since stem cell quality is critical for the differentiation of these cells. iPSCs with PB-TAD-7F are closer to the map of chicken ESCs, therefore, the quality of iPSCs with PB-TAD-7F would be higher than those with PB-R6F. Based on those results, we chose iPSCs expressing PB-TAD-7F to form organoids in this study. The critical difference between iPSCs with PB-TAD-7F and iPSCs with PB-R6F is the overexpression of *Klf2* [11, 12]. Takashima et al. reported that overexpression of *Klf2* could lead to a naïve-like state in human-primed stem cells [29]. We, therefore, considered that iPSCs with PB-TAD-7F might improve stem cell quality compared to iPSCs with PB-R6F, although both iPSCs with PB-R6F and iPSCs with PB-TAD-7F are primed state iPSCs.

According to the stem cell quality, mammalian iPSCs are characterized into two types: naïve state (e.g., mouse iPSCs) and primed state (e.g., human iPSCs). Naïve state iPSCs are more advantageous as a bio-resource since their differentiation ability is higher than that of primed state iPSCs. Changes in the iPSCs from primed to naïve states are not understood in avian species. This study listed the metabolism-related pathway through DEGs analysis between iPSCs with PB-TAD-7F and Stage X embryo-derived cells. These results might provide clues on changing the nature of chicken iPSCs from a primed state to a naïve state.

Using methods developed in the human model, we attempted to establish organoids containing neural-like cells from chicken pluripotent stem cells [17, 18]. These methods were serum-free for the maturation of neural organoids. In contrast to human pluripotent stem cells, the serum is essential for the self-renewal of chicken iPSCs [11, 12]. We therefore hypothesized that serum concentration might critically affect the maturation of chicken iPSC-derived organoids containing neural-like cells. Based on that hypothesis, we investigated serum concentration for the maturation of chicken organoids. In this study, 2% FBS medium was the most well-differentiated to neural-like cells compared with 10% serum, 5% serum, and serum-free media. Therefore, we concluded that a low-serum medium would be useful for the maturation of chicken iPSC-derived organoids containing neural-like cells compared with 5 and 10% serum medium.

Poly:IC can stimulate RLR members such as *MDA5* [24, 30]. Therefore, we tried to evaluate the function of *MDA5* and *LGP2* with poly:IC exposure. *MDA5* and *LGP2* expression in organoids significantly increased after poly: IC exposure. In addition to *MDA5* and *LGP2*, *IL-6*, *IL-1 beta*, and *Mx1* expression also increased after 6 h of exposure to poly:IC. These genes are stimulated by RLR-derived signals [23]. Based on these results, we concluded that organoids containing neural-like cells respond to poly:IC through *MDA5* and *LGP2*. Therefore, we considered that organoids containing neural-like cells would respond to the innate immunity to viral RNA through the RLR families. The exposure of poly:IC would require more work to evaluate whether our organoids containing neural-like cells can use to evaluate viral infectious disease since poly:IC is an analog of viral RNA. In the next study, we will expose those viruses to evaluate whether our organoids recognize the lethal virus.

In addition to 6 h of exposure to poly:IC, *MDA5*, *LGP2*, *IL1beta*, and *Mx1* mRNA levels increased in cut organoids at 24 h compared to control levels (S2a Fig in S1 File). In contrast to organoid cutting methods, the trypsinization of organoids resulted in cell death by this method and did not increase the mRNA levels of RLRs (*MDA5* and *LGP2*) after 24 h of exposure to poly:IC (S2b Fig in S1 File). Therefore, we concluded that the cut organoids could recognize poly:IC at multiple time points.

Influenza is a well-known lethal infectious disease caused by RNA viruses. More than 400,000 non-poultry birds, including wild birds, died of infectious avian influenza virus (AIV) between 2021 and 2022 worldwide [14]. Various avian species such as white-tailed sea eagles and African Penguins have been reported to cause influenza infections [31, 32]. Therefore, AIV are important infectious diseases for the conservation of endangered species. A chicken study reported that neurological symptoms occurred with AIV [33–35]. Furthermore, in chickens and ducks, the brain is listed as having one of the most prominent lesions upon histological analyses [36]. In addition to histological analysis, AIV RNA has been detected in chicken, turkey, and duck brains [36]. In white-tailed sea eagles, it is suggested that the severity of influenza is attributable to viral replication in the brain [31]. Based on these reports, neural cells are useful in predicting the risk of mortality in endangered avian species. In this study, we successfully generated organoids containing neural-like cells from chicken iPSCs. iPSCs can be established from dead, individually derived somatic cells. Therefore, iPSCs can be established from endangered avian species. We have already established endangered avian-derived iPSCs [12]. We plan to expand this technique to the formation of organoids of endangered species.

The development of organoid technology allows the reproduction of tissues and organs; therefore, several medical and pharmaceutical scientists have focused on this technology [37]. Although the nature of organoids does not perfectly agree with that of tissues and organs in vivo, their nature is similar to that of tissues and organs in 2D cultured cells. Several organoids have been used to study human infectious diseases. For example, human lung organoids have been used for the study of influenza [38], and human brain organoids have been used to study Zika virus infection [39]. In addition to infectious diseases, human kidney organoids have also been used for nephrotoxicity tests [40]. Brain, pancreas, and stomach organoids are used in developmental biology [41–43]. In contrast to human studies, organoid technology has not yet been developed for other species. Therefore, we believe that our results will provide useful information for tissue and cellular engineering as well as for animal, livestock, and veterinary scientists.

Our method may be economically expensive; therefore, there is room for improvement. In future studies, we intend to address the development of low-cost methods. The establishment term needs to be shortened because we needed 50–53 days for the maturation of organoids in this study. We plan to focus on the temperature during the formation of organoids because the body temperature of avian species is higher than that of mammals; for example, the chicken body temperature is 41 °C [44]. We hypothesized that avian species-derived organoids might undergo high-speed formation with the optimization of temperature.

In this study, chicken iPSC-derived organoids containing neural-like cells responded to poly:IC via RLR. In a recent study by our group, we established iPSCs from endangered avian species-derived somatic cells [12]. These iPSCs can differentiate into various cells as chicken iPSCs. Therefore, we consider that endangered avian species-derived organoids containing neural-like cells could also be formed from these iPSCs. In the future, we believe these organoids could be developed as a new evaluation tool for infectious diseases in non-model avian species, including endangered avian species.

## Conclusion

To the best of our knowledge, this is the first report on forming organoids containing neural-like cells from chicken iPSCs. Our organoids containing neural-like cells responded to poly:IC via RLR. In this study, we developed iPSCs technology for avian species via organoid formation. We believe that organoids containing neural-like cells from avian iPSCs can be developed as a new evaluation tool for infectious disease risk in avian species, including endangered species.

## Supporting information

S1 File (DOCX)
