# Supplementary material for: Organoids containing neural-like cells derived from chicken iPSCs respond to poly:IC through the RLR family
Source: PLoS One. 2023 May 4;18(5):e0285356. doi: 10.1371/journal.pone.0285356 (PMC10159107; doi:10.1371/journal.pone.0285356)
Supplement: S1 File — (DOCX) [file pone.0285356.s001.docx]

**Supplementary information**

Organoids containing neural-like cells derived from chicken iPSCs respond to poly:IC through the RLR family

Masafumi Katayama, Manabu Onuma, Noriko Kato, Nobuyoshi Nakajima, Tomokazu Fukuda.


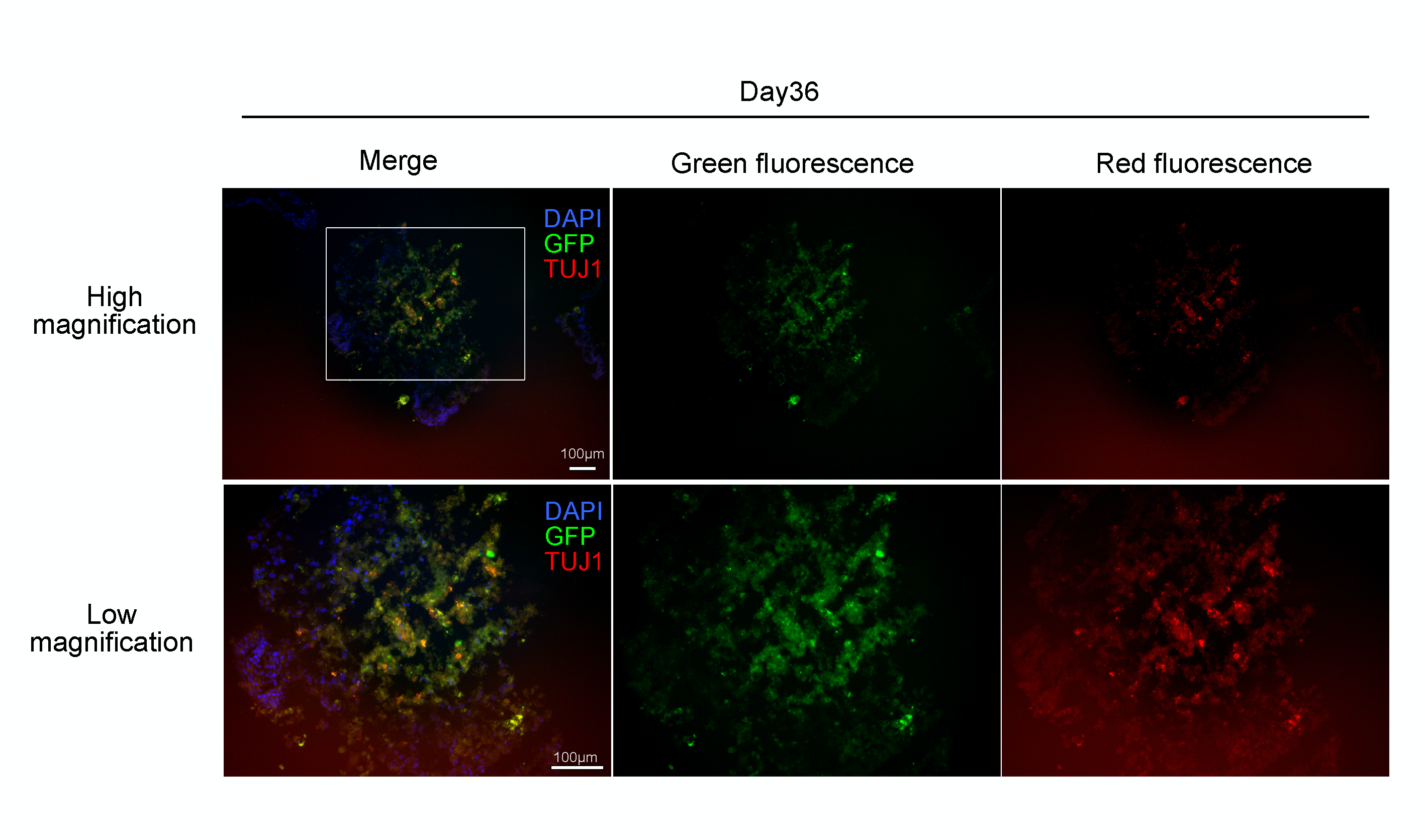


**S1 Fig. Immunological staining of chicken organoids containing neural-like cells.**

Panels shod the merged image (left), the green fluorescence (middle), and red fluorescence (right). Uppers are high magnification, lowers are low magnification. Bars represent 100 μm.


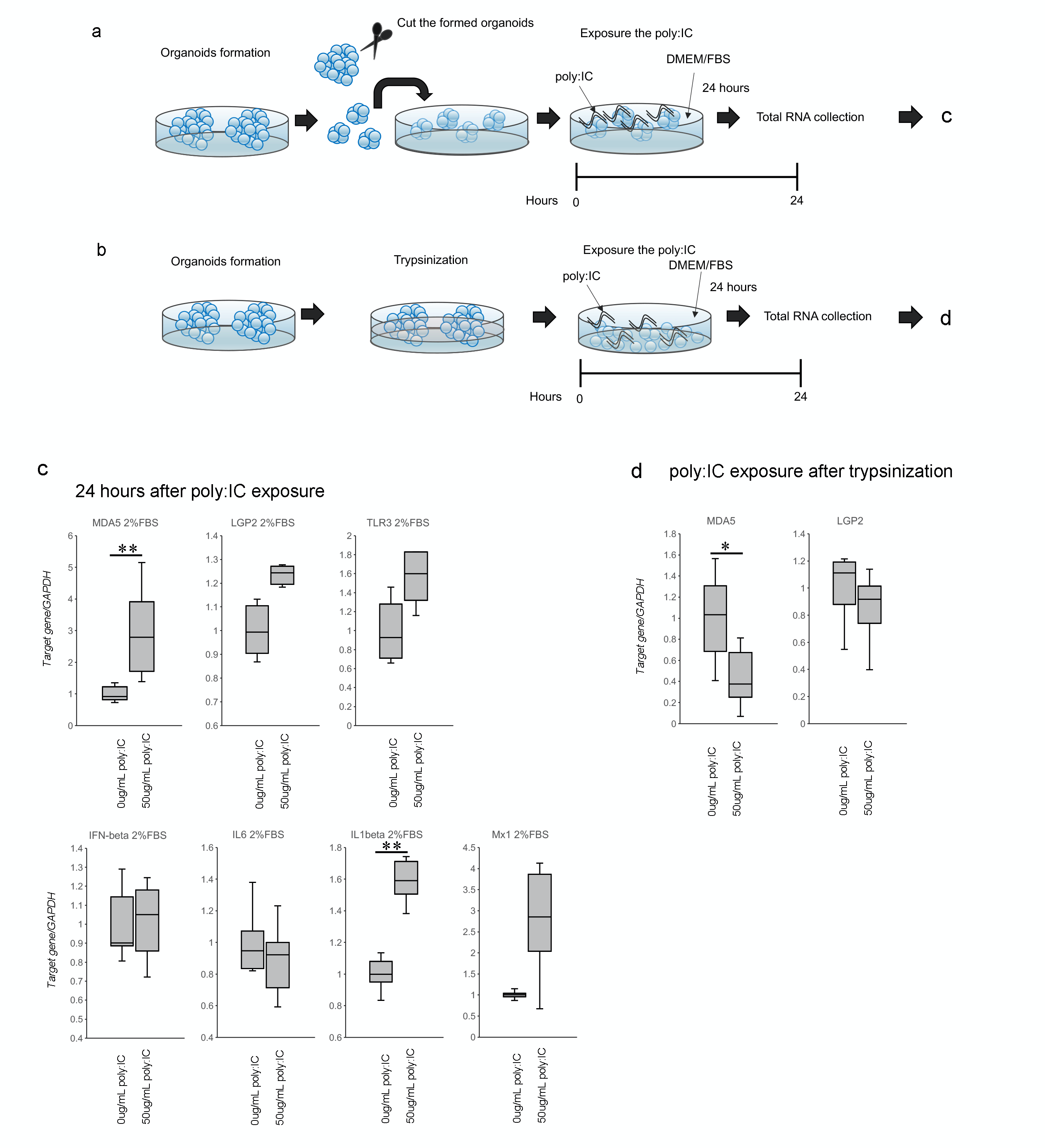


**S2 Fig. Gene expression in chicken organoids containing neural-like cells in 2% serum medium for maturation after 24 h of polyIC exposure.** Gene expression of *MDA5, LGP2, TLR3, IFN-beta, IL-6, IL-1 beta,* and *Mx1* in cut organoids (a). Gene expression of *MDA5*,and *LGP2* in trypsinization organoids (b). The left bars show control, the right bars show exposure to poly:IC 50 μg/mL. Centerlines of box plots indicate medians; box limits indicate 25th and 75th percentiles. n=6. * P<0.05. Gene expression was quantified relative to the *GAPDH* internal control. The control expression was set at 1.0.

S1 Table Primer sequence of chicken qPCR.

S2 Table. Chi-square test.
